# Research on Positioning and Navigation System of Greenhouse Mobile Robot Based on Multi-Sensor Fusion

**DOI:** 10.3390/s24154998

**Published:** 2024-08-02

**Authors:** Bo Cheng, Xueying He, Xiaoyue Li, Ning Zhang, Weitang Song, Huarui Wu

**Affiliations:** 1 College of Water Resources and Civil Engineering, China Agricultural University, Beijing 100083, China; bs20243090921@cau.edu.cn (B.C.);; 2 College of Horticulture, Henan Agricultural University, Zhengzhou 450002, China; 3 National Engineering Research Center for Information Technology in Agriculture, Beijing 100097, China

**Keywords:** agricultural greenhouse, navigation robot, multi-sensor fusion, ultra-wideband, inertial measurement unit, odometry, rangefinder

## Abstract

The labor shortage and rising costs in the greenhouse industry have driven the development of automation, with the core of autonomous operations being positioning and navigation technology. However, precise positioning in complex greenhouse environments and narrow aisles poses challenges to localization technologies. This study proposes a multi-sensor fusion positioning and navigation robot based on ultra-wideband (UWB), an inertial measurement unit (IMU), odometry (ODOM), and a laser rangefinder (RF). The system introduces a confidence optimization algorithm based on weakening non-line-of-sight (NLOS) for UWB positioning, obtaining calibrated UWB positioning results, which are then used as a baseline to correct the positioning errors generated by the IMU and ODOM. The extended Kalman filter (EKF) algorithm is employed to fuse multi-sensor data. To validate the feasibility of the system, experiments were conducted in a Chinese solar greenhouse. The results show that the proposed NLOS confidence optimization algorithm significantly improves UWB positioning accuracy by 60.05%. At a speed of 0.1 m/s, the root mean square error (RMSE) for lateral deviation is 0.038 m and for course deviation is 4.030°. This study provides a new approach for greenhouse positioning and navigation technology, achieving precise positioning and navigation in complex commercial greenhouse environments and narrow aisles, thereby laying a foundation for the intelligent development of greenhouses.

## 1. Introduction

Greenhouses provide crops with suitable microclimates to mitigate the impact of external natural climates on agriculture. Greenhouse cultivation enables off-season vegetable production, enhances crops’ resistance to pests and diseases, and improves both the quality and yield of crops to meet the growing demand for vegetables [1,2]. However, challenges such as labor shortages and rising costs restrict the development of greenhouses [3]. To reduce reliance on manual labor and achieve greenhouse automation [4], fully autonomous operation has become the primary solution. Precise positioning and navigation, as the core technology for autonomous operation, have become critical challenges that urgently need to be addressed for the intelligent development of greenhouses.

A greenhouse is characterized by a large steel framework and densely grown crops inside, resulting in significant differences between the greenhouse environment and the outdoor environment during navigation and positioning [5,6]. Autonomous navigation technologies for greenhouses mainly include fixed rail, simultaneous localization and mapping (SLAM), and track-following systems [7]. Fixed rail navigation, including ground-based and suspended types, requires the installation of tracks within the greenhouse, which will affect other production processes in the greenhouse [8]. SLAM is primarily divided into visual SLAM [9,10] and laser SLAM [11,12], which involve map construction followed by path planning to achieve navigation [13,14,15,16]. Path planning includes global path planning and local path planning [17]. Global planning, a form of static planning, calculates the optimal path from start to end points based on the known layout of the environment, often utilizing algorithms such as A* [18,19] and ant colony [20,21] optimization. Local planning, a form of dynamic planning, updates map information in real time based on sensor data to generate a local optimal path from the current node to the next sub-goal node, commonly employing techniques like artificial potential fields [22] and neural network algorithms [23]. Due to the high similarity in texture and structure of greenhouse environments, as well as the dense foliage obstructing visibility, feature extraction based on visual and laser methods becomes challenging [24,25], rendering such navigation technologies less applicable for greenhouses. Tracked navigation includes wired and wireless tracking methods. The former relies on magnetic strips [26], QR code tags, or wires for guidance, offering limited flexibility. Wireless track navigation utilizes technologies like ultra-wideband (UWB) positioning to determine navigation paths. UWB technology, with its advantages of a high bandwidth, short pulses, and strong penetration capabilities, significantly reduces the impact of obstacles on pulse signals compared to other wireless communication technologies. Therefore, it is more suitable for navigation and positioning applications within greenhouses [27,28,29]. A single sensor’s signal penetration capability is affected by complex channels, making it difficult to accurately perceive the movement characteristics of mobile objects [30]. Similarly, positioning accuracy relying solely on UWB is also limited under conditions with numerous non-line-of-sight (NLOS) environments indoors in greenhouses [31,32,33].

The main influencing factor of UWB positioning accuracy is NLOS effects. Naheem et al. [34] proposed an improved loosely coupled Kalman filtering algorithm that integrates UWB and an IMU into a positioning system. This system not only eliminates the IMU’s cumulative error drift but also filters out the NLOS error effects of UWB. Compared to traditional data fusion positioning systems, the positioning accuracy is improved by 27%. Liu et al. [35] utilized altitude information provided by a barometer to mitigate NLOS effects on UWB. By employing Kalman filtering (EKF) to fuse UWB and IMU information, the positioning accuracy increased by 45.71%. Sun et al. [36] employed a method combining power gain and probability statistics to identify NLOS signals, using an inertial navigation system to compute UWB position information, achieving indoor positioning in NLOS environments. However, the impact of NLOS effects in greenhouse environments remains unknown.

Compared with a single sensor, the fusion of multi-sensor information can provide a more comprehensive and accurate perception of the motion characteristics of moving objects [37,38,39]. Based on UWB technology, integrating other sensors for auxiliary positioning has become one of the important methods for solving greenhouse autonomous navigation. Zhang [40] proposed a positioning method that integrates UWB and laser LiDAR, with an average error that is reduced from 32 cm with UWB alone to 7 cm after fusion, demonstrating that multi-sensor fusion can effectively enhance navigation accuracy. Bi et al. [41] designed a fusion method using an extended Kalman filter for UWB ranging correction values and inertial measurement unit (IMU) data, achieving a positioning error of 11.95 cm under NLOS communication conditions. Long et al. [42] introduced a multi-sensor fusion positioning method combining UWB, an IMU, wheel odometry (ODOM), and laser LiDAR, achieving an error of 7.9 cm in positioning. However, greenhouse environments impose stringent requirements on navigation accuracy, particularly in narrow aisle planting greenhouses, where existing research struggles to meet navigation and positioning demands.

To enhance the accuracy of greenhouse positioning and navigation, this study proposes a navigation robot that integrates UWB, an IMU, ODOM, and a rangefinder (RF) for sensor fusion, aiming to overcome individual sensor limitations. Based on this, an algorithm is proposed to mitigate NLOS confidence for UWB specifically tailored for greenhouse environments, thereby improving the accuracy of positioning and navigation. To verify the feasibility and precision of this navigation robot, experiments will be conducted within a Chinese solar greenhouse located in Beijing, China to assess the positioning effectiveness and navigation accuracy during the robot’s movement.

## 2. Positioning System of Mobile Robot Based on Multi-Sensor Fusion

### 2.1. Multi-Sensor Fusion Positioning Method

**Figure 1:**
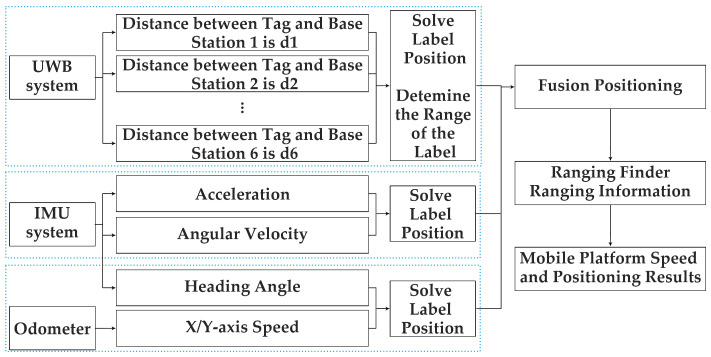
A fusion positioning framework based on UWB/IMU/ODOM/RF.

The multi-sensor fusion positioning system comprises UWB, an IMU, an odometer, and an RF. The UWB positioning tag, IMU, odometer, and RF are all installed on the mobile robot. The UWB system calculates the position information of the mobile robot through the distance between the tag and each positioning base station and combines the heading angle of the mobile robot output by the IMU device and the linear speed of the vehicle body in the X and Y directions obtained by the odometer. The obtained data are fused using the EKF algorithm and combined with the data obtained from the rangefinder. The principle is shown in Figure 1.

### 2.2. UWB Positioning Model Optimization

**Figure 2:**
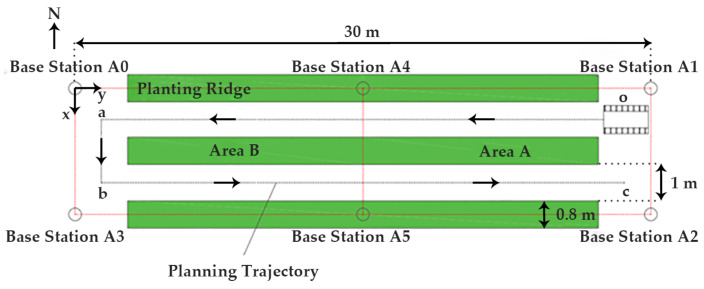
Layout of UWB positioning base station and planning trajectory.

According to the principles of positioning in three-dimensional space, obtaining precise coordinate information typically requires at least four positioning base stations. Due to the high planting density inside greenhouses, navigation systems encounter extensive NLOS conditions. Therefore, the number of base stations in the UWB positioning system is increased to six, and the positioning area is divided into two equally sized regions (as shown in Figure 2). Each region forms a calibration system composed of four positioning base stations and one mobile tag. In the basic UWB positioning algorithm, the ranging information from each base station carries equal weight, and the computed position in unobstructed environments can be considered the actual position. However, in greenhouse environments, each base station is affected by NLOS to varying degrees. Therefore, it is necessary to assess the extent of NLOS’s impact on the ranging values of each base station. Based on these assessments, different weights are assigned to the ranging values of affected base stations: those less affected are given greater weights, while those more affected are given lower weights. This approach aims to improve the accuracy of position calculations from the UWB positioning system in greenhouse conditions.

#### 2.2.1. Determine NLOS Environments

The method proposed in this study to identify the NLOS environment is to compare the abscissa position of the mobile robot at a certain moment calculated by the measurement value of the laser rangefinder with the abscissa position calculated by the UWB positioning system, thereby determining whether the mobile robot is affected by NLOS. Therefore, a laser rangefinder is introduced into the original positioning system. The laser rangefinder selected in this article has a measurement accuracy of ±1.5 cm in short-distance mode. We use the distance between the mobile robot and the ridges on both sides measured by the laser rangefinder to calculate the abscissa *C_m_* of the mobile robot in the navigation coordinate system at *k* time:

(1) $$C{\left(k\right)}_{m}=l+L+\frac{x_{0}-h\cos\alpha_{0}}{\sin\alpha_{0}}$$

It can be seen from Figure 2 that the mobile robot forms a “U-shaped” trajectory within the radiation range of the base station. Therefore, when the mobile robot travels to an aisle far away from the base station A0, the abscissa calculated by Formula (1) needs to be increased by a fixed value, which is as follows:

(2) $$C{\left(k\right)}_{m}=l+L+\frac{x_{0}-h\cos\alpha_{0}}{\sin\alpha_{0}}+1.8$$

In the formula, *l* is the distance between the laser ranging finder and the vehicle UWB tag; *L* is the distance from the bottom of the ridge to the base station A0; *x*_0_ is the perception distance of the ranging finder; *α*_0_ is the bottom angle of the ridge; *h* is the height of the laser ranging finder from the ground; and 1.8 is the sum of the ridge and aisle widths.

Compare with the abscissa *C_UWB_* obtained by the UWB system and calculate the difference at *k* time determined as follows:

(3) $$\left|\Delta C\left(k\right)\right|=\left|C{\left(k\right)}_{UWB}-C{\left(k\right)}_{m}\right|$$

Select an appropriate judgment threshold *γ* to judge whether each base station is in the non-line-of-sight environment of the mobile robot.

#### 2.2.2. Weakening the Impact of NLOS

If |ΔC(k)|<3γ, it is determined that the mobile robot is in the line-of-sight (LOS) environment at this time. That is, the coordinate position obtained by the UWB positioning system is reliable, and there is no need to weaken the influence of NLOS and continue to execute the driving program.

If |ΔC(k)|≥3γ, the position information calculated by the UWB positioning system is affected by NLOS, so the obtained fused positioning information is inaccurate, and the influence of NLOS needs to be weakened. Firstly, the operating area of the mobile robot is determined according to the coordinate position of the tag to be tested and calculated by the UWB system. It can be seen from Figure 2 that when the mobile robot is driving in the aisle, no matter where it is in any position in any area, there are at least two base stations in the LOS environment. At this time, it is only necessary to weaken the influence of NLOS on the other two base stations. Secondly, we combine the base station affected by NLOS with the two base stations in the LOS environment. We calculate the difference between the lateral position of the tag under the test measured by two different base station combinations and the lateral position calculated by the ranging finder. Based on this difference, the confidence *β_i_* of the base station ranging value in the NLOS environment can be determined as follows:

(4) $$|\beta_{i}=1/D_{Li}|$$

In the formula, *D_Li_* is the lateral position difference of the tag to be tested determined by different base station combinations.

Then, we use the laser ranging finder installed at the same height as the UWB tag on the vehicle to measure the height h of the tag from the ground at this time and calculate the height difference Δ*h* between the UWB base station and the tag. Then, the horizontal distance *d_i_* between the tag to be tested and each base station is derived from the ranging value *d_mi_* between the tag to be tested and each base station:

(5) $$d_{i}=\sqrt{d_{mi}^{2}-\Delta h^{2}}$$

Finally, we incorporate the confidence level and use the least squares method to solve the estimated coordinate value of the label to be tested at this time.

We compare the UWB abscissa information after weakening the influence of NLOS with the abscissa of the mobile robot calculated from the ranging information again. If |ΔC(k)|<3γ, the positioning information after weakening the influence of NLOS can meet the requirements and continue to execute the driving program. If |ΔC(k)|≥3γ, the abscissa in the navigation coordinate system obtained by using the weakening algorithm cannot meet the requirements. At this time, the abscissa calculated by the ranging finder and the ordinate calculated by the UWB positioning system are combined to form the coordinate information of the mobile robot.

Although the positioning accuracy of the UWB system in the greenhouse is lower than that of the laser rangefinder, due to the poor greenhouse environment, if the mobile robot uses the abscissa calculated by the rangefinder throughout its movement, the trajectory of the mobile robot will be a wave. Therefore, only when the accuracy of other sensors is affected, the positioning data of the rangefinder will be fused, ensuring that the mobile robot can walk straight.

### 2.3. IMU Error Judgment and Compensation

When the IMU works for a long time, it is easy to generate cumulative errors, affecting the accuracy of the proposed positioning system. This study employs the following methods to assess and compensate for its cumulative errors.

After the above calibration process, the UWB system can obtain more accurate coordinate information. At this time, the cumulative error of the IMU can be determined and compensated for based on the UWB positioning results. The coordinate value calculated by the UWB is compared with the coordinate value calculated by the IMU device to obtain *|δP|* as follows:

(6) $$|\delta P|=|P_{UWB}-P_{IMU}|$$

If the difference is more significant than *3γ* but |*ΔC(k)*| < 3*γ*, it means that the UWB positioning result is more accurate, while the IMU positioning result is less so. At this time, the positioning result of the UWB system can be used to compensate for the IMU error, and the specific value *δR(k)* that needs to be compensated for the error at time *k* is calculated by Formula (6):

(7) $$\delta R(k)=\left|P{\left(k\right)}_{UWB}-P{\left(k\right)}_{IMU}\right|$$

Then the position information calculated by the IMU system at the subsequent *k +* 1 time can be calibrated with this value:

(8) $$P_{\mathrm{adj}}{\left(k+1\right)}_{IMU}=P{\left(k+1\right)}_{IMU}-\delta R(k)$$

### 2.4. Odometer Positioning Analysis

#### 2.4.1. Establish the Kinematics Model

**Figure 3:**
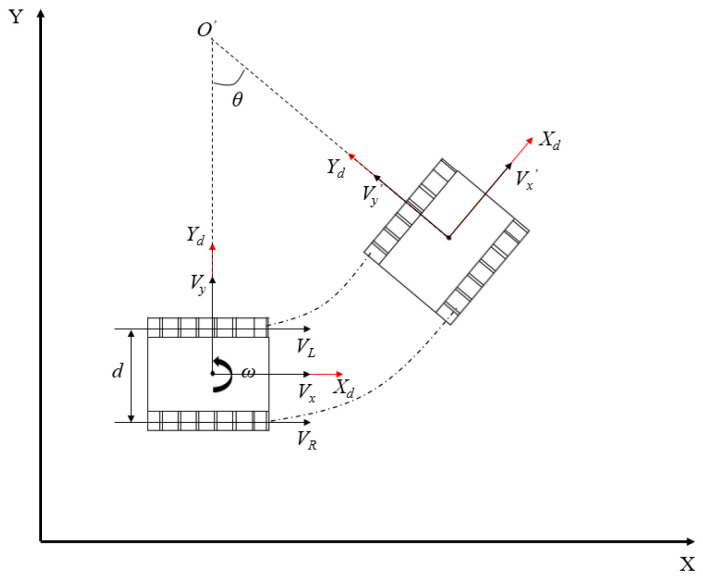
Schematic diagram of mobile robot’s kinematics.

The greenhouse mobile robot adopts a crawler chassis. For the convenience of our calculations, the kinematics model can be simplified as a two-wheel differential movement model, and only the movement in two-dimensional space is considered. The kinematics model of the mobile robot in the aisle is shown in Figure 3.

According to the wheel speed measured by the encoders of the two driving wheels of the mobile robot and the kinematics model constructed, the whole vehicle’s linear velocity and angular velocity can be calculated. In adjacent time intervals, it can be considered that the mobile robot moves in a circle with *O’* as the center, and the rotation angle is *θ*. It is considered that the two-wheel differential chassis does not need to consider lateral slippage, so the mobile robot in the carrier coordinate system can obtain the expressions of linear velocity and angular velocity as follows:

(9) $$\left[\begin{array}{c} V_{y}\\ \omega \end{array}\right]=\left[\begin{array}{cc} \frac{1}{2} & \frac{1}{2}\\ \frac{1}{d} & -\frac{1}{d} \end{array}\right]\left[\begin{array}{c} V_{L}\\ V_{R} \end{array}\right]$$

In the formula, *V_y_* is the linear velocity of the mobile robot along the *Y_d_* axis; *V_L_* and *V_R_* correspond to the linear velocity of the left and right wheels of the mobile robot, respectively; *ω* is the rotational angular velocity of the mobile robot in a two-dimensional plane; and *d* is the wheelbase of the left and right wheels. We can obtain the motion equation of the mobile robot in the global coordinate system as follows:

(10) $$\left[\begin{array}{c} \overset{\cdot }{X}\\ \overset{\cdot }{Y}\\ \overset{\cdot }{\theta } \end{array}\right]=\left[\begin{array}{ccc} \cos\theta & -\sin\theta & 0\\ \sin\theta & \cos\theta & 0\\ 0 & 0 & 1 \end{array}\right]\left[\begin{array}{c} V_{x}\\ V_{y}\\ \omega \end{array}\right]$$

#### 2.4.2. Odometer Error Correction

The mobile robot uses two photoelectric encoders to form an odometer positioning system. However, the driving wheels will slip to a certain extent when turning in place in the solar greenhouse on non-hardened roads, resulting in inaccurate attitude angles calculated through the odometer. The pose error calculated in Formulas (9) and (10) will accumulate with time, so the positioning accuracy obtained by this method will decrease [43].

This study employs orientation sensing provided by an IMU to assist in the attitude detection during the steering process of the mobile robot. Angular velocity is utilized as a system variable to compare the angular velocity computed from odometry with that sensed by the IMU, thereby determining the slipping condition of the robot’s two tracks. In cases of slipping, the heading angle information provided by the IMU is used as observed angular data to steer the mobile robot.

To address the cumulative positioning errors of odometry, this study adopts a feature point correction method. When odometry accumulates errors, it leads to deviations in the position of the mobile robot. This position becomes a feature point, which is corrected using fused information from UWB and rangefinders to address the cumulative errors in odometry. The longitudinal position information of the mobile robot provided by UWB and the lateral position information obtained from rangefinders are integrated to establish the initial position of the odometer, enabling the continuation of navigation and positioning tasks in the subsequent stages.

### 2.5. Multi-Sensor Fusion Algorithm

#### 2.5.1. Fusion of UWB/IMU/ODOM Data Based on Extended Kalman Filter

This study integrates UWB, an IMU, and ODOM under NLOS conditions, all of which are nonlinear. The extended Kalman filter (EKF) effectively handles nonlinear models using linearization techniques, while maintaining a relatively low level of computational complexity, making it suitable for real-time positioning in resource-constrained embedded systems [44,45]. Therefore, this study employs the EKF to fuse data from multiple sensors. 

We assume the operating environment of the mobile robot is a two-dimensional plane in an ideal state. The pose of the mobile robot is used as the state vector in the fusion algorithm, and the data information provided by the UWB and IMU is used for measurement updates. We establish the state model and measurement model of the combined positioning system as follows:

(11) $$\{\begin{array}{c} x_{k+1}=f(x_{k},u_{k+1})+w_{k+1}\\ z_{k+1}=h(x_{k+1})+v_{k+1} \end{array}$$

In the formula, *x_k+_*_1_ is the state variable at time *k +* 1; *u_k+_*_1_ is the control variable at time *k +* 1; *f(·)* is the functional relationship between the state variable *x_k+_*_1_ and the state variable *x_k_*; *w_k+_*_1_ is the motion noise at time *k +* 1; *z_k+_*_1_ is the system observation value at time *k +* 1; and *v_k+_*_1_ is the observation noise at time *k +* 1. The pose information *u_k+_*_1_ *=* [*V_O,X_, V_O,Y_, ω_O_*] calculated by the odometer is used as the control amount in the prediction stage, where *V_O,X_, V_O,Y_,* and *ω_O_*, respectively, represent the movement speed of the mobile robot in the *X*-axis direction and the *Y*-axis direction in the navigation coordinate system and the heading angle of the mobile robot.

According to the established kinematic model of the mobile robot, the pose at *k +* 1 time can be expressed as follows:

(12) $${\overset{\wedge -}{x}}_{k+1}={\overset{\wedge }{x}}_{k}+\left[\begin{array}{ccc} \cos\theta_{k} & -\sin\theta_{k} & 0\\ \sin\theta_{k} & \cos\theta_{k} & 0\\ 0 & 0 & 1 \end{array}\right]\left[\begin{array}{c} V_{O,X}\\ V_{O,Y}\\ \omega_{O} \end{array}\right]dt$$

The covariance matrix of the system state quantity in the prediction stage at time *k +* 1 is as follows:

(13) $$P_{k+1}^{-}=f_{x}P_{x}f_{x}^{T}+f_{w}Q_{k+1}f_{w}^{T}$$

In the formula, *P_k+_*_1_ is the covariance matrix of the state quantity *x_k+_*_1_; and *Q_k+_*_1_ is the covariance matrix of the motion noise of the wheel odometer.

The Jacobian matrix of the constructed kinematic model and the Jacobian matrix of motion noise are, respectively, as follows:

(14) $$fx=\left[\begin{array}{ccc} 1 & 0 & (-V_{X}\sin\theta_{k}+V_{Y}\cos\theta_{k})dt\\ 0 & 1 & (V_{X}\cos\theta_{k}-V_{Y}\sin\theta_{k})dt\\ 0 & 0 & 1 \end{array}\right]$$

(15) $$f_{w}=\left[\begin{array}{ccc} (\cos\theta_{k})dt & (-\sin\theta_{k})dt & 0\\ (\sin\theta_{k})dt & (\cos\theta_{k})dt & 0\\ 0 & 0 & 1 \end{array}\right]$$

The difference between the heading angle information *θ_k+_*_1_ provided by the IMU at time *k +* 1 and the coordinate position information provided by UWB and the position information calculated by the IMU is input into the system as the observation value, and we obtain the observation model at this time as follows:

(16) $$z_{k+1}=\left[\begin{array}{c} P_{adj}{\left(k+1\right)}_{IMU,x}-P{\left(k+1\right)}_{UWB,x}\\ P_{adj}{\left(k+1\right)}_{IMU,y}-P{\left(k+1\right)}_{UWB,y}\\ \theta_{k+1} \end{array}\right]+v_{k+1}$$

The calculated Kalman gain coefficient is as follows:

(17) $$K_{k+1}=P_{k+1}^{-}H_{x}^{T}{\left(H_{x}P_{k+1}^{-}H_{x}^{T}+R_{k+1}\right)}^{-1}$$

In the formula, *H_x_* is the Jacobian matrix of the observation model; *R_k+_*_1_ is the covariance matrix of the observation noise; and the noise estimate determined according to the sensor accuracy given by the manufacturer is as follows:

(18) $$R_{k+1}=\left[\begin{array}{ccc} \sigma_{X} & 0 & 0\\ 0 & \sigma_{Y} & 0\\ 0 & 0 & \sigma_{\theta } \end{array}\right]$$

In the formula, *σ_X_* is the observation noise variance in the UWB output in the *X*-axis direction; *σ_Y_* is the observation noise variance in the UWB output in the *Y*-axis direction; and *σ_θ_* is the observation noise variance in the attitude angle output by the IMU.

The posterior estimation of the state vector is as follows:

(19) $${\overset{\wedge }{x}}_{k+1}={\overset{\wedge -}{x}}_{k+1}+K_{k+1}(z_{k+1}-{\overset{\wedge -}{x}}_{k+1})$$

Substituting the posterior estimate of the state vector into the covariance matrix of the prediction stage yields a covariance matrix with posterior estimates:

(20) $$P_{k+1}=(I_{3}-K_{k+1})P_{k+1}^{-}$$

In the formula, *I*_3_ is the third-order identity matrix.

In summary, the fusion of UWB, IMU, and wheel odometer data has been achieved. However, among the three sensors, UWB is susceptible to the influence of NLOS, and it is difficult to meet the high-precision requirements, while both the IMU and wheel odometers have the disadvantage of error accumulation.

#### 2.5.2. Correcting the Position of the Mobile Robot Based on the Ranging Value

To compensate for the cumulative errors in UWB, the IMU, and wheel odometry, rangefinders are employed as auxiliary sensors. The lateral coordinates of the mobile robot are calculated using the ranging methods described in Formulas (1) and (2), and these coordinates are utilized to compensate for positioning errors caused by external influences and inherent inaccuracies in the other three sensors.

The mobile robot drives along the aisle in the greenhouse, forming a “U-shaped” trajectory. When the mobile robot reaches the set target point, it will stop moving and then execute commands to go to the next target point. And when the robot starts, the timestamp at this time and the length information on both sides of the distance sensed by the rangefinder will be recorded as reference data when subsequent large lateral deviations occur in the mobile robot.

When the UWB is affected by NLOS, and the IMU [46] and the odometer system have a significant degree of deviation due to the accumulation of errors, resulting in a sizeable lateral offset of the mobile robot, the system will automatically cut off the sampling update of the IMU and the odometer. At the same time, the lateral position information provided by the ranging finder is compared with the data of the starting position, and the deviation between the two is used as the input value. The PID algorithm is used to drive the mobile robot back to the established channel, and then the position information calculated by UWB, the IMU, and the odometer is integrated into the system.

## 3. Experiments for Verification

### 3.1. Experimental Greenhouse and Mobile Robot Details

#### 3.1.1. Experimental Greenhouse

**Figure 4:**
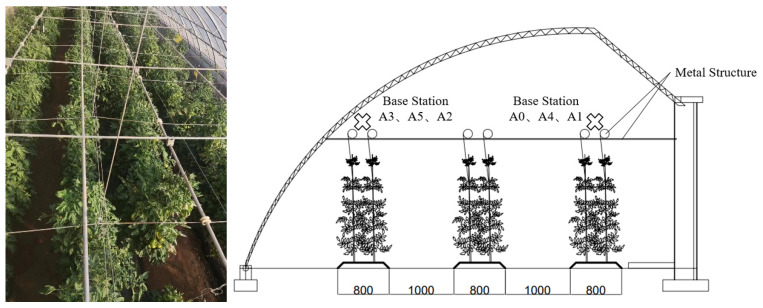
Greenhouse environment.

To validate the feasibility of this positioning and navigating robot, experiments were conducted in a solar greenhouse located in Beijing, China. The experimental greenhouse measures 30 m in length (east to west) with a span of 5 m (north to south). The crops are planted in east–west rows. The planting ridges are 0.8 m wide with aisles that are 1 m wide. The actual conditions inside the greenhouse are depicted in Figure 4.

#### 3.1.2. Mobile Robot Description

**Figure 5:**
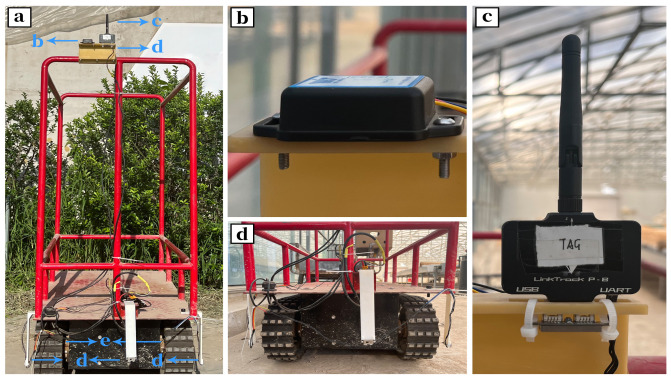
Greenhouse mobile robot ((**a**)—overall robot; (**b**)—inertial measurement unit; (**c**)—UWB tags; (**d**)—rangefinder; (**e**)—odometer).

The mobile robot is mainly designed to meet the requirements of the confined environment of a Chinese solar greenhouse (CSG) with sufficient power and flexibility. The dimensions of the mobile robot are designed to be 1 m in length, 0.6 m in width, and 1.5 m in height (including the protective frame), with a track width of 0.1 m (as shown in Figure 5). The mobile chassis is equipped with a 48 V 80 Ah lithium battery and two 48 V 1700 W brushless DC reduction motors. The main control chip is STM32H750VBT6, powered separately by a 7.4 V 1500 mAh lithium battery. UWB positioning tags and IMU devices are installed on the top of the mobile robot’s cargo platform, 1.5 m above the ground, to minimize environmental signal interference. At the same height, an RF facing the ground is installed to assist the UWB positioning algorithm. The odometer is installed at the position of the drive wheel, coaxial with it, to record the changes in the distance moved relative to the ground and the directional angle of the wheel. Three RF modules are installed on both sides and directly in front of the mobile robot, working with other sensors to achieve precise positioning navigation and obstacle avoidance.

### 3.2. Experimental Study on Static Positioning Accuracy of UWB in NLOS Environments

To validate the feasibility of the NLOS optimization algorithm mentioned earlier, the corridor was partitioned into sections with 5 m intervals. The midpoint of each corridor section at the end of the region served as the test point for UWB positioning accuracy. After manually moving the robot to the test point, it stops to collect positioning data, then it is moved to the next test point under the control of the operator.

### 3.3. Navigation Accuracy Experiment

The mobile robot departs from the starting point o at (1.25 m, 28 m), sequentially passing through the three target points: a at (1.25 m, 1.5 m), b at (4 m, 1.5 m), and c at (4 m, 28 m), as shown in Figure 2. Testing occurs at intervals of 2 m within the aisle and at intervals of 1 m along the path from a to b.

**Figure 6:**
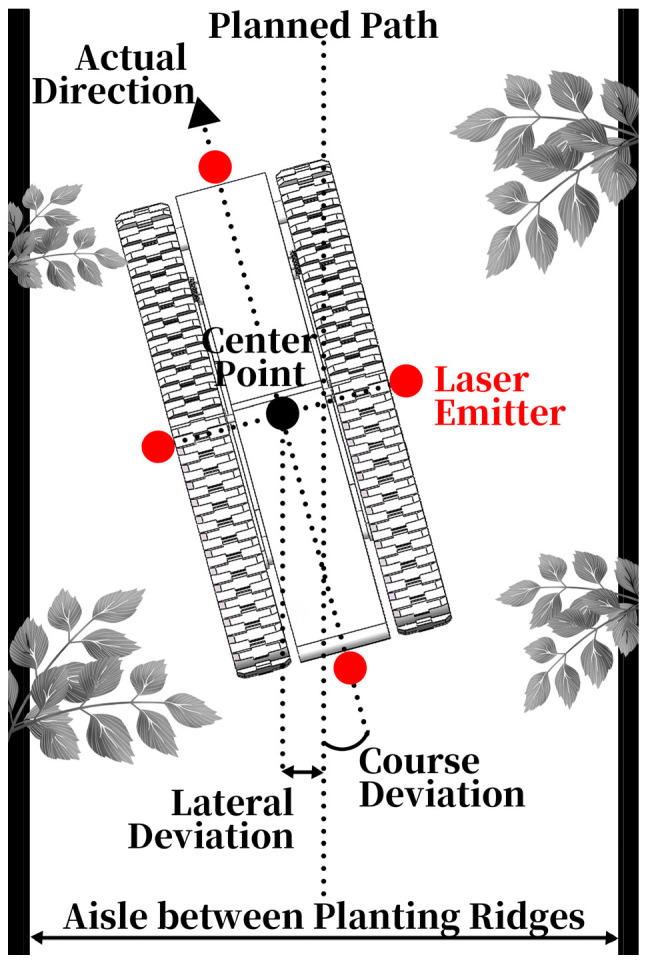
Metrics for navigation accuracy measurement.

To assess the navigation accuracy of a mobile robot during its operation, laser measurement is employed. A laser emitter, facing the ground, is installed at the midpoint around each side of the navigation robot (as shown in Figure 6). Upon reaching the test point, markings from the four laser emitters are recorded. The intersection point formed by the cross of the four marked points is considered the center point. The perpendicular distance from this center point to the planned path represents the lateral deviation of the navigation robot (as shown in Figure 6). The angle between the longitudinal axis of the navigation robot and the planned path constitutes the course deviation (as shown in Figure 6). Lateral deviation and course deviation serve as quantitative indicators of navigation accuracy. The navigation robot is programmed to stop for 10 s and record deviations every 2 m along the planned path.

A comparison of lateral deviations between the sensor measurement method and the aforementioned laser measurement method is shown in Table 1. The root mean square error (RMSE) of lateral deviation by the sensor measurement method is 0.070 m, while for the laser measurement method, the RMSE of lateral deviation is 0.040 m. Therefore, this study utilizes the laser measurement method to assess the navigation accuracy of the mobile robot.

## 4. Results and Discussion

### 4.1. Analysis of UWB Static Localization Results in NLOS Environments

The coordinates of 10 test points where UWB tags were statically positioned along with the coordinate data before and after the attenuation of NLOS’s effects and their root mean square errors are presented in Table 2. It can be seen from Table 2 that the minimum value of the root mean square error before weakening is 0.244 m, the maximum value is 0.497 m, and the average positioning error is 0.398 m in the static positioning test under the NLOS environment; the minimum value of the root mean square error is 0.115 m, the maximum value is 0.226 m, and the average positioning error is 0.159 m after the algorithm proposed in this paper weakens the influence of NLOS. It can be found that the UWB positioning system is affected by NLOS, so the positioning accuracy cannot meet the requirements of precise positioning, and the weakening algorithm proposed in this paper improves the UWB positioning accuracy by 60.05%. The impact of NLOS has a good weakening effect.

### 4.2. Analysis of Navigation Accuracy

The navigation accuracy test was carried out according to the route planned in Figure 2. The deviation data obtained at each test point are shown in Table 3. It is stipulated that the mobile robot is positive when facing left, and it is positive when it deviates from the trajectory to the left. As shown by the test data in Table 3, at a speed of 0.1 m/s, the average lateral deviation of the mobile robot is 0.037 m, the RMSE is 0.038 m, the average course deviation is 3.557°, and the RMSE is 4.030°. However, the maximum lateral deviation of two adjacent test points is 0.095 m, and the maximum course deviation is 9.663°. The larger deviations appear near the initial movement positions of the north and south aisles and the alternate positions of areas A and B. This phenomenon is because the vehicle body of the mobile robot is not oriented correctly at the initial position, and the deviation correction of the system lags, causing the mobile robot to produce relatively large corrections. In addition, the alternate positions of area A and area B are affected by NLOS, which makes the position of the tag to be tested calculated by the UWB positioning system inaccurate, and it is impossible to accurately determine which area the mobile robot is in. And the IMU and the odometer have a large cumulative error after a long work period. At this time, the ranging finder can only be used to correct the position of the mobile robot, which affects the accuracy of data fusion. However, when the mobile robot continues to drive for a certain distance, it can run smoothly and precisely when its direction is corrected and the sensor information is re-integrated into the system.

To test the overall navigation effect of the mobile robot, the mobile robot is continuously driven according to the previously planned path, and the lateral deviation of the driving track is judged according to the wheel marks on the driving path according to a distance that is twice the vehicle’s length. The test data are shown in Table 4. The data shown in Table 4 show that the proportion of lateral deviations between 0.03 m and 0.04 m is the largest, and the proportion of deviations between 0.02 m and 0.05 m is as high as 86.67%. It can be seen that the fusion positioning and navigation system can be used in solar greenhouses with complex environments, effectively improving positioning and navigation accuracy.

## 5. Conclusions and Prospects

A navigation robot utilizing fused positioning with UWB, an IMU, ODOM, and an RF was proposed, with an optimized UWB algorithm tailored for greenhouse conditions. Our experimental validation demonstrated the feasibility and accuracy of the navigation robot. The main conclusions of this study are as follows: 

A weighted positioning algorithm is proposed to mitigate the effects of NLOS environments on UWB localization results. The results show that compared to before optimization, the proposed algorithm improves the robot’s positioning accuracy by 60.05%, demonstrating its ability to enhance the localization precision of navigation robots in greenhouse environments.

We form a greenhouse environment positioning and navigation system set based on UWB, an IMU, an odometer, and a ranging finder. As UWB, the IMU, and the odometer are affected by the external environment and their cumulative errors in the greenhouse, we aimed to reduce the positioning accuracy. The ranging data information is introduced into the multi-sensor fusion system, and our experiments verify the multi-sensor fusion system. When the mobile robot is driving at a speed of 0.1 m/s, the average lateral deviation is 0.037 m, the RMSE is 0.038 m, the average course deviation is 3.557°, and the RMSE is 4.030°, which further improve the navigation accuracy.

The fusion technology of UWB, an IMU, ODOM, and an RF proposed in this study provides a new approach for greenhouse positioning and navigation technology, achieving precise positioning and navigation in complex commercial greenhouse environments and narrow aisles. This lays a foundation for the intelligent development of greenhouses. However, NLOS environments vary among different greenhouses and crops. Therefore, it is necessary to explore more signal processing methods under different NLOS conditions in further research to enhance the robustness and accuracy of UWB positioning.

## Figures and Tables

**Table 1:** Comparison of lateral deviation measured by two methods.

	Sensor Measurement Deviation/m	Laser Measurement Deviation/m
2 m	0.074	0.028
4 m	0.082	0.049
6 m	0.088	0.037
8 m	0.089	0.036
10 m	0.094	0.031
12 m	0.028	0.052
14 m	0.047	0.036
16 m	0.070	0.047
18 m	0.074	0.038
20 m	0.085	0.034
22 m	0.047	0.039
24 m	0.025	0.046
26 m	0.056	0.043

**Table 2:** Static positioning results of UWB positioning system in NLOS environment.

Actual Coordinates/m	Average Positioning Coordinates/m		RMSE/m	
	Before Weakening	After Weakening	Before Weakening	After Weakening
(1.250, 25.000)	(1.283, 25.251)	(1.252, 25.231)	0.244	0.126
(1.250, 20.000)	(1.236, 20.430)	(1.240, 20.350)	0.261	0.209
(1.250, 15.000)	(1.334, 15.164)	(1.254, 15.160)	0.481	0.115
(1.250, 10.000)	(1.521, 10.473)	(1.252, 10.274)	0.458	0.141
(1.250, 5.000)	(1.334, 5.382)	(1.248, 5.152)	0.329	0.119
(4.000, 5.000)	(4.249, 5.596)	(4.016, 5.402)	0.497	0.226
(4.000, 10.000)	(4.138, 10.472)	(4.065, 10.302)	0.400	0.201
(4.000, 15.000)	(4.451, 15.292)	(3.987, 15.176)	0.457	0.122
(4.000, 20.000)	(4.143, 20.625)	(4.012, 20.196)	0.407	0.140
(4.000, 25.000)	(3.744, 25.477)	(4.009, 25.324)	0.447	0.189

**Table 3:** Navigation accuracy test results.

Coordinate Test Point/m	Lateral Deviation/m	Course Deviation/°
(1.25, 26.00)	−0.043	1.426
(1.25, 24.00)	−0.046	2.103
(1.25, 22.00)	0.039	−3.277
(1.25, 20.00)	−0.034	1.325
(1.25, 18.00)	−0.038	1.019
(1.25, 16.00)	−0.047	−1.715
(1.25, 14.00)	0.036	−4.179
(1.25, 12.00)	−0.052	3.332
(1.25, 10.00)	0.031	1.415
(1.25, 8.00)	0.036	2.927
(1.25, 6.00)	−0.037	−4.348
(1.25, 4.00)	−0.049	−2.246
(1.25, 2.00)	0.028	−5.433
(2.25, 1.50)	−0.024	3.586
(3.25, 1.50)	0.031	−2.729
(4.00, 2.00)	−0.052	2.331
(4.00, 4.00)	0.043	−2.211
(4.00, 6.00)	0.049	5.226
(4.00, 8.00)	0.033	−3.846
(4.00, 10.00)	−0.043	−4.233
(4.00, 12.00)	−0.048	−6.817
(4.00, 14.00)	0.022	1.215
(4.00, 16.00)	−0.032	−6.424
(4.00, 18.00)	0.027	3.239
(4.00, 20.00)	0.022	−3.622
(4.00, 22.00)	−0.031	−7.391
(4.00, 24.00)	−0.033	−8.143
(4.00, 26.00)	0.035	−3.835

**Table 4:** Navigation accuracy test results.

Absolute Range of Lateral Deviation/m	Proportion/%
(0, 0.01]	0
(0.01, 0.02]	0
(0.02, 0.03]	26.67
(0.03, 0.04]	40
(0.04, 0.05]	20
(0.05, 0.06]	6.67
(0.06, 0.07]	3.33
(0.07, 0.08]	0
(0.08, 0.09]	3.33
(0.09, 0.10]	0

## Data Availability

Data are contained within the article.
